# Traumatic and stressful life events as precipitants of obsessive compulsive disorder and social anxiety disorder

**DOI:** 10.1002/jts.70021

**Published:** 2025-11-12

**Authors:** Verônica Hühne, Samara dos Santos‐Ribeiro, Maria Eduarda Moreira‐de‐Oliveira, Carla P. Loureiro, Gabriela B. de Menezes, Leonardo F. Fontenelle

**Affiliations:** ^1^ Anxiety, Obsessions and Compulsions Research Program Institute of Psychiatry Federal University of Rio de Janeiro Rio de Janeiro Brazil; ^2^ D'Or Institute for Research and Education Rio de Janeiro Brazil; ^3^ Department of Psychiatry School of Clinical Sciences Monash University Clayton Australia

## Abstract

Potentially traumatic events (PTEs) and stressful life events (SLEs) are recognized as environmental risk factors for diverse psychiatric disorders, including obsessive compulsive disorder (OCD) and social anxiety disorder (SAD). However, research has predominantly focused on the presence and quantity of PTE/SLE exposure rather than specific event types or associated emotions. This study aimed to investigate the role of PTEs/SLEs in the onset of OCD and SAD. We recruited patients diagnosed with OCD (*n* = 38) or SAD (*n* = 25) and contrasted their responses to the Childhood Trauma Questionnaire–Short Form (CTQ‐SF) and the newly developed Obsessive‐Compulsive Related Disorders Stressful and Traumatic Events Scale (OTraS), which was built to measure events related to OCD and related disorders. Data analysis was performed using Mann–Whitney tests. Childhood trauma severity did not differ between groups; however, OTraS responses demonstrated that participants with OCD reported exposure to a significantly higher number of PTEs/SLEs, *r* = .25, *p* = .044, especially those related to loss and deprivation, than those with SAD, *r* = .36; *p* = .004. Importantly, this difference remained significant after controlling for the presence of hoarding disorder, *p* = .012. Our findings indicate that PTEs/SLEs, particularly those that were loss‐ and deprivation‐related, are more common before OCD onset than SAD onset. Further research is needed to explore whether different PTE/SLE types are transdiagnostically relevant for the whole spectrum of OCD‐ and anxiety‐related disorders or may shape specific disorder development in at‐risk individuals.

The relationship between obsessive compulsive disorder (OCD) and social anxiety disorder (SAD) is multifaceted and has evolved over time, reflecting shifting theoretical, diagnostic, and empirical perspectives. In the third edition of the *Diagnostic and Statistical Manual of Mental Disorders* (*DSM*‐*III*; American Psychiatric Association [APA], 1980), both OCD and SAD were categorized under the umbrella of anxiety disorders. With the release of the *DSM‐5* (APA, 2013), a significant nosological shift occurred: OCD was reclassified under a newly created chapter titled “obsessive‐compulsive and related disorders” (OCRDs), whereas SAD remained within anxiety disorders.

Despite their diagnostic distinction, evidence suggests some degree of clinical and etiological overlap between OCD and SAD, contributing to an ongoing sense of ambiguity regarding their separation. In the recent World Mental Health Surveys, SAD was found to predict OCD onset, severity, and treatment‐seeking (Stein et al., 2025). Additionally, our research has shown that subthreshold symptoms of OCD, SAD, and panic disorder (PD) are highly correlated in milder cases and tend to differentiate into distinct phenotypes as symptom severity increases (Vigne et al., 2019). This phenomenon aligns with the pluripotent model of at‐risk mental states in the context of other disorders, which suggests that early, nonspecific psychopathology may evolve into distinct disorders over time (Hartmann et al., 2021).

This raises a critical question: If OCD and SAD are so closely intertwined in early stages, what drives their divergence into more specific and distinct clinical syndromes? Environmental factors likely play a pivotal role in this transition. Although both OCD and anxiety disorders have been associated with exposure to stressful life events (SLEs) and potentially traumatic events (PTEs), the specific role of adverse experiences in shaping diagnostic trajectories remains unclear. By identifying links between specific diagnoses and PTE/SLE exposure, clinicians may be better equipped to identify at‐risk populations and tailor psychoeducation and treatment accordingly. Notably, to our knowledge, no prior study has directly compared the prevalence and thematic content of PTEs/SLEs in individuals with OCD versus those with SAD. To address this gap, we developed the Obsessive‐Compulsive Related Disorders Stressful and Traumatic Events Scale (OTraS), a novel instrument designed to assess life events thought to be etiologically relevant to OCD and related disorders (Fontenelle, Destrée, et al., 2021).

In the present study, we compared the prevalence and nature of PTE/SLE exposure in individuals with a primary diagnosis of OCD versus SAD. To do so, we used both a general measure of childhood adverse experiences and the OTraS. We hypothesized that the OTraS would be able to identify higher rates of symptom‐relevant life events among individuals with OCD and SAD. In contrast, we posited that the traditional childhood trauma questionnaire would lack the discriminative validity to differentiate between these two groups.

## METHOD

### Participants

This study is part of the Transdiagnostic Predictors of Response to Treatment with Serotonin Reuptake Inhibitors (TransPoRT) study. The TransPoRT study was a naturalistic study conducted to evaluate predictors for response to treatment with serotonin reuptake inhibitors (SRIs). Patients were recruited from a specialized treatment center for OCD and SAD at the Federal University of Rio de Janeiro. Eligibility was defined according to four medication scenarios: (a) initiation of an SRI, at the treating physician's discretion, in patients either untreated or receiving non‐SRI medications; (b) dose adjustment to the maximum tolerated level in patients receiving subtherapeutic SRI doses; (c) maintenance of a therapeutic SRI dose in patients treated for fewer than 12 weeks at baseline; and (d) switching to another SRI in patients with inadequate response to an adequate treatment.

The inclusion criteria comprised (a) being at least 18 years old, (b) having OCD or SAD as the most relevant diagnosis (i.e., the more severe clinical condition and, for this reason, most influential in guiding the decision to initiate or adjust pharmacotherapy), (c) being at least “moderately ill” according to the Clinical Global Impression (CGI; i.e., having a CGI of at least 4; Guy, 1976) and (d) being able to read and fill out forms. Researchers, who were trained postgraduate students, assessed patients using the Mini‐International Neuropsychiatric Interview (MINI; Sheehan et al., 1998) to confirm their diagnosis. As the MINI does not include a specific module for hoarding disorder, hoarding symptoms, including judgments regarding clinical relevance (i.e., the presence of hoarding disorder), were assessed through a detailed review of clinical notes. The research team analyzed the notes written by the assistant physician and extracted diagnostic and symptom‐related information. The research was conducted in accordance with the ethical standards of the Research Ethics Committee of the Institute of Psychiatry at the Federal University of Rio de Janeiro, which approved the research protocol (CAAE: 26274019.7.0000.5263). Eligible participants were informed of the study's goals and invited to participate after providing a written consent form.

### Measures

#### Emotional distress

The Depression, Anxiety, and Stress Scales–Short Form (DASS‐21; P. Lovibond & Lovibond, 1995; S. Lovibond. & Lovibond, 1995) is a self‐report measure consisting of 21 items that assesses past‐week symptoms of emotional distress across three subscales: Depression, Anxiety, and Stress. Each subscale comprises seven items that are rated on a 4‐point Likert scale ranging from 0 (*never*) to 3 (*almost always*), with scores summed and higher scores indicating higher symptom levels both overall (total scale) and for each subscale. The DASS‐21 has been validated in Brazilian Portuguese (Vignola & Tucci, 2014), and this version has demonstrated good psychometric properties, including strong internal consistency (Cronbach's alpha = .90 for the Depression subscale, .86 for the Anxiety subscale, .88 for the Stress subscale, and .95 for the total scale). In the present study, the subscales also showed strong internal consistency, with Cronbach's alpha values of .88 for Depression, .85 for Anxiety, and .90 for Stress.

#### Childhood trauma exposure

The Childhood Trauma Questionnaire–Short Form (CTQ‐SF; Bernstein et al., 2003; Hagborg et al., 2022) is a 28‐item self‐report instrument that retrospectively assesses the frequency of adverse and potentially traumatic childhood events in individuals who are at least 12 years old. Of the 28 items, 25 assess maltreatment, and three detect minimization or the denial of maltreatment. The CTQ‐SF measures five dimensions of maltreatment: emotional, physical, and sexual abuse, as well as emotional and physical neglect. For these items, responses are rated on a 5‐point Likert scale ranging from 1 (*never true*) to 5 (*very often true*), with higher scores indicating higher degrees of trauma exposure. The minimization and denial items only consider the highest positive score (5). The CTQ‐SF is a validated scale with an average Cronbach's alpha of .89 for the total score. Reported values for the subscales range from .66 for Physical Neglect to .92 for Sexual Abuse, and test–retest reliability for the total score was found to be .79 (Badenes‐Ribera et al., 2024). In this study, the CTQ‐SF was utilized to quantify childhood trauma severity in all participants. The subscales demonstrated adequate‐to‐strong internal consistency, with Cronbach's alpha values of .72 for Emotional Neglect, .85 for Emotional Abuse, .87 for Physical Abuse, and .85 for Sexual Abuse. In contrast, the physical neglect subscale yielded poor internal consistency, Cronbach's α = .31.

#### Traumatic and stressful life events relevant to OCD and related disorders

The OTraS (Fontenelle, Destrée, et al., 2021) is a self‐report inventory designed to assess the occurrence of PTEs/SLEs before the onset of a given disorder. The scale lists 60 potential PTEs/SLEs thought to be related to different OCRDs symptoms, including themes of harm (e.g., physical aggression), sex or immorality (e.g., sexual abuse), contamination (e.g., contact with dangerous chemicals or toxins), symmetry (e.g., being punished or humiliated for being disorganized), physical appearance (i.e., bullying over one's physical imperfections), and loss and deprivation (e.g., extreme financial difficulty or food insecurity), with scores ranging from 0 (*no events*) to 10 (*maximum events*) for each theme. The first four themes reflect events thought to be related to OCD, the fifth theme to body dysmorphic disorder, and the last theme to hoarding disorder.

Only events that (a) the participant perceived as significantly traumatic or stressful and (b) happened before symptom onset were recorded, as many events may have occurred but been regarded as emotionally standard or neutral. In addition, participants were asked to rate the intensity of six emotions (fear, horror, or helplessness; disgust; rage; guilt; shame; and sadness) on a scale of 1 (*least intense*) to 5 (*most intense*), as experienced during their most significant PTE/SLE. Through the OTraS, it is possible to quantify (a) the total number of PTEs/SLEs related to OCRDs in general, (b) the number of PTEs/SLEs related to each OCRD theme, and (c) the intensity of each emotion associated with the most stressful event.

### Data analysis

We compared the prevalence of these PTE/SLE themes between the OCD and SAD groups, controlling for the presence of hoarding symptoms as documented in participants’ clinical charts. Data analyses were performed using SPSS (Version 29). Mann–Whitney tests were conducted, with CTQ‐SF subscale scores and the number of PTEs/SLEs in the OTraS (both total and by theme) as dependent variables and diagnostic group as the independent variable. Statistical significance was defined as a *p* value less than .05. Effect sizes (*r*) were calculated using Cohen's formula (Cohen, 2013; Fritz et al., 2012) and interpreted as small (0.1–0.3), medium (0.3–0.5), or large (> 0.5). To assess whether hoarding acted as a confounding variable, we additionally performed an analysis of covariance (ANCOVA). There were no missing data among the 63 participants included in the analysis.

## RESULTS

### Description of the sample

A total of 63 adults completed data collection. The mean participant age was 37.5 years. Regarding gender, 33 (52.4%) participants were female, and 30 (47.6%) were male. Regardless of the primary diagnosis, the majority lived with their parents and siblings (22.2%), identified as White (65.1%), were employed (49.2%), and had completed higher education (54.0%). According to the MINI, patients were diagnosed with OCD (*n* = 38) or SAD (*n* = 25) as their most clinically relevant diagnostic condition (see Table 1). Based on chart review, only four participants likely had hoarding disorder, all of whom were in the OCD group.

**TABLE 1:** Sample description

Variable	OCD (*n* = 38)		SAD (*n* = 25)		Total (*n* = 63)	
	*M*	*SD*	*M*	*SD*	*M*	*SD*
Age (years)	39.21	12.1	34.84	13.2	37.50	12.6

	***n***	**%**	***n***	**%**	***n***	**%**
Female gender	18	47.4	15	60.0	33	52.4
Other individuals in the household						
Parent and sibling(s)	16	42.1	10	40.0	26	41.2
Alone	5	13.2	3	12.0	8	12.7
Spouse (with or without children)	10	26.3	8	32.0	18	28.6
Other	7	18.4	4	16.0	11	17.5
Race/ethnicity						
Caucasian	28	73.7	13	52.0	41	65.1
Black	3	7.9	2	8.0	5	7.9
Mixed race	6	15.8	9	36.0	15	23.8
Other	1	2.6	1	4.0	2	3.2
Educational attainment						
Primary education	3	7.9	1	4.0	4	6.3
Secondary education	16	42.1	9	36.0	25	39.7
Higher education	19	50.0	15	60.0	34	54.0
Work status						
Employed	18	47.4	13	52.0	31	49.2
Unemployed	7	18.4	4	16.0	11	17.5
Retired or on leave due to disability	4	10.5	2	8.0	6	9.5
Other	9	23.7	6	24.0	15	23.8

*Note*: OCD = obsessive‐compulsive disorder; SAD = social anxiety disorder

### Emotional distress

There were no significant differences in the severity of symptoms of emotional distress between the OCD and SAD groups as measured using the DASS‐21. The groups did not differ significantly in any of the subscales (see Supplementary Table S1).

### PTE/SLE exposure

There were no significant differences in childhood trauma severity between the OCD and SAD groups as measured using the CTQ‐SF. None of the assessed categories showed significant between‐group differences (see Table 2). On the OTraS, 60 participants reported exposure to at least one PTE/SLE, with a maximum of 37 events per participant recorded (*M* = 13.3) out of the 60 assessed events. The events were categorized into six themes, each comprising up to 10 possible events. Participants reported a mean of 2.2 harm‐related events (range: 0–6), 1.6 sexual events (range: 0–5), 2.6 contamination‐related events (range: 0–9), 2.3 symmetry‐related events (range: 0–9), 2.3 body dysmorphia–related events (range: 0–9), and 2.3 hoarding‐ or loss and deprivation–related events (range: 0–8).

**TABLE 2:** Between‐group differences in Childhood Trauma Questionnaire–Short Form (CTQ‐SF) scores

CTQ‐SF subscale	OCD (*n* = 38)		SAD (*n* = 25)			
	*M*	*SD*	*M*	*SD*	*r*	*p*
Emotional Abuse	6.16	4.79	5.12	3.96	.08	.525
Physical Abuse	2.61	3.80	1.76	3.68	.18	.132
Sexual Abuse	0.89	1.77	1.48	2.49	.05	.626
Emotional Neglect	14.76	3.91	16.24	5.36	.18	.151
Physical Neglect	5.71	2.03	6.44	2.97	.13	.299

*Note*: OCD = obsessive compulsive disorder; SAD = social anxiety disorder.

Participants in the OCD group reported exposure to significantly more PTEs/SLEs than those in the SAD group, *r* = .25, *p* = .044, particularly with regard to experiences of loss and deprivation, *r* = .36, *p* = .004. This difference remained significant even after controlling for probable hoarding disorder, *p* = .012. No significant between‐group differences were found for other PTE/SLE themes, including harm, sex and morality, contamination, symmetry, or body dysmorphia (see Table 3). Additionally, there were no significant between‐group differences in emotional responses (e.g., shame, fear, horror, helplessness, disgust, rage, guilt, and sadness) related to the most significant PTE/SLE experience (see Supplementary Table S2).

**TABLE 3:** Between‐group differences in the prevalence of potentially traumatic and stressful life event (PTE/SLE) themes

PTE/SLE theme	OCD (*n* = 38)		SAD (*n* = 25)			
	*M*	*SD*	*M*	*SD*	*r*	*p*
Harm	2.37	1.67	1.92	1.41	.12	.340
Sexual	1.61	1.48	1.56	1.39	.00	.994
Contamination	3.05	2.54	1.96	1.95	.21	.093
Symmetry	2.82	2.63	1.44	1.29	.23	.064
Hoarding (loss and deprivation)	2.82	1.89	1.48	1.66	.36	.004
Body dysmorphia	2.58	1.93	1.88	1.54	.17	.176
All	15.24	9.05	10.24	6.06	.25	.044

*Note*: OCD = obsessive‐compulsive disorder; SAD = social anxiety disorder.

## DISCUSSION

SLEs and PTEs are highly prevalent among individuals with psychiatric disorders, as reflected in our sample, in which only three of the 63 total participants reported no PTE/SLE exposure before the onset of their anxiety disorder. The near‐universal presence of these events in our sample underscores their importance as the focus of research aimed at understanding their potential role in the onset, symptom severity, and treatment response of psychiatric conditions. Given the high prevalence of PTE/SLE exposure, the routine assessment of life events in clinical practice should be encouraged (Boals, 2018). These events can serve as specific topics for psychological interventions, particularly those based on acceptance and mindfulness (Ojserkis et al., 2020).

We found a higher prevalence of PTE/SLE exposure in the OCD group compared to the SAD group, as measured using the OTraS but not the CTQ‐SF. Although extensive research has examined the role of SLEs as risk factors for psychiatric disorders by comparing clinical samples with healthy controls (Khanna et al., 1988; McKeon et al., 1984; Sarkhel et al., 2011), few studies have compared their pre–symptom onset prevalence across different disorders. De Loof et al. (1989) and Gothelf et al. (2004) reported a higher prevalence of SLEs in OCD compared to PD and other anxiety disorders. Our findings add to this evidence by showing that SLE exposure that occurred before symptom onset is more frequent in OCD than in SAD. However, given that the studies used different measures of SLE assessment, the generalizability remains limited.

Importantly, childhood trauma of sustained and prolonged nature has been associated with the development of complex posttraumatic stress disorder (CPTSD; Giourou et al., 2018). Although evidence indicates that both childhood trauma and CPTSD symptoms may influence the course and severity of OCD (D'Angelo, Valenza, Iazzolino, Longobardi, Di Stefano, Visalli, et al., 2024), PD (D'Angelo, Valenza, Iazzolino, Longobardi, Di Stefano, Lanzara, et al., 2024), and SAD, our study did not determine whether the reported traumatic events were persistent in nature, nor did we assess the presence of PTSD or CPTSD within the sample. The emerging transdiagnostic literature suggests that this association could be a valuable direction for future research.

Examining specific PTE/SLE themes, we found that loss and deprivation–related events more frequently preceded the onset of OCD than SAD, even when controlling for comorbid hoarding symptoms. This suggests that specific PTE/SLE themes may be more etiologically relevant to OCD than to other disorders. Other potentially relevant factors influencing the impact of PTEs/SLEs include the individual's subjective perception of trauma and stress (Boals, 2018), coping style (Elderton et al., 2017), and emotional resilience (Hühne et al., 2021). Notably, SLEs have been previously reported among self‐identified hoarders (Fontenelle, Muhlbauer, et al., 2021) and have been linked to hoarding disorder (Landau et al., 2011). However, most studies have not distinguished whether these events occurred before or after symptom onset (Cromer et al., 2007; Tolin et al., 2010), which limits conclusions about their role as precipitating or maintaining factors for hoarding. Identifying risk factors for psychiatric disorders is essential for informing clinicians about at‐risk populations and for guiding targeted therapeutic interventions.

Beyond the small sample size, another key limitation of this study is the absence of a distinct hoarding disorder comparison group, which would have allowed for an evaluation of whether there is a higher prevalence of loss and deprivation–related PTEs/SLEs among individuals with a primary diagnosis of hoarding disorder compared to those with OCD. We did, however, control for the presence of hoarding, which was unable to eliminate the association between OCD and loss and deprivation–related PTE/SLE exposure. We admit, however, that chart reviews may not be enough to identify hoarding disorder, which has symptoms that often go unnoticed in both OCD and anxiety samples (Tolin et al., 2011). Future research exploring the role of different PTE/SLE themes in the onset of various psychiatric disorders could improve understanding of these events as risk factors and their contribution to the pathophysiology of mental disorders, both individually and across diagnoses.

Although previous studies have emphasized the role of interpersonal trauma in OCD, such as sexual and body dysmorphia–related events (Ojserkis et al., 2020), our findings suggest that noninterpersonal events, such as loss and deprivation, may also be relevant and warrant further investigation. Nevertheless, in our study, the impact of interpersonal trauma in OCD may have been less apparent due to the comparison with SAD, which is also strongly associated with interpersonal events (Marteinsdottir et al., 2007). Consequently, the role of interpersonal events in OCD may have been less distinguishable in this context. Future research should compare OCD with other clinical samples to determine whether our findings are consistent across different populations.

Another limitation of this study is its reliance on self‐reported, retrospective assessments of PTEs, SLEs, and associated emotions, which may introduce memory biases, such as overestimation or omission of certain events and/or peritraumatic emotions. Additionally, the OTraS depends on the participant's subjective perception of an event as stressful or traumatic, which may affect consistency. However, a strength of our study is that the OTraS minimizes heterogeneity, as it only assesses events that participants perceive as stressful or traumatic, ensuring the inclusion of events with a genuine psychological impact (Pinciotti & Fisher, 2022).

Our findings suggest that PTEs/SLEs, particularly those related to loss and deprivation, as measured by the OTraS and not the CTQ‐SF, are more prevalent before the onset of OCD than the onset of SAD. This indicates that an OCD‐specific scale may be necessary to better identify relevant PTEs/SLEs in this population. Further research is needed to determine whether specific PTE/SLE themes have transdiagnostic relevance across the whole spectrum of OCD and anxiety–related disorders or contribute to the emergence of distinct conditions in at‐risk individuals. Additionally, studies with nonclinical samples are essential to understand the protective factors in individuals who experience PTEs/SLEs but do not develop a psychiatric disorder, providing insight into their role in mental health outcomes. Given their clinical significance, PTEs/SLEs should be routinely assessed in clinical practice, as they may serve as potential targets for psychological interventions, particularly those based on acceptance and mindfulness. Further research is warranted to explore the role of PTEs/SLEs as therapeutic targets.

## AUTHOR CONTRIBUTIONS

**Verônica Hühne**: Conceptualization; writing ‐ original draft; methodology; writing review & editing; software; investigation; data curation; formal analysis. **Samara dos Santos‐Ribeiro**: Conceptualization; investigation; methodology; supervision; data curation; project administration. **Maria Eduarda Moreira‐de‐Oliveira**: Conceptualization; investigation; methodology; data curation; supervision; project administration. **Carla P. Loureiro**: Conceptualization; investigation; data curation; methodology. **Gabriela B. de Menezes**: Conceptualization; writing ‐ review & editing; methodology; supervision; project administration. **Leonardo F. Fontenelle**: Project administration; conceptualization; methodology; writing ‐ review & editing; supervision.

## AUTHOR NOTE

Gabriela B. de Menezes and Leonardo F. Fontenelle were co–last authors.

## OPEN PRACTICES STATEMENT

The study reported in this article was not formally preregistered. Neither the data nor the materials have been made available on a permanent third‐party archive; requests for the data or materials can be sent via email to the corresponding author at lfontenelle@gmail.com.

## Supporting information



Supporting Information
