# Supplementary material for: Traumatic and stressful life events as precipitants of obsessive compulsive disorder and social anxiety disorder
Source: J Trauma Stress. 2025 Nov 12;39(1):151–8. doi: 10.1002/jts.70021 (PMC12890773; doi:10.1002/jts.70021)
Supplement: Supplementary file 1 — Supporting Information [file JTS-39-151-s001.docx]

***Table S1****: Severity of Comorbidity Symptoms*

|  | **OCD**  ***(n = 38)*** | | **SAD**  ***(n = 25)*** | |  |  |
| --- | --- | --- | --- | --- | --- | --- |
| **DASS-21** | **Mean** | **SD** | **Mean** | **SD** | ***Effect Size (r)*** | ***P-value*** |
| Depression | 18.11 | 10.39 | 19.68 | 12.20 | *.04* | *.741* |
| Anxiety | 14.79 | 10.35 | 12.72 | 11.24 | *.13* | *.321* |
| Stress | 21.68 | 10.56 | 20.80 | 11.08 | *.06* | *.652* |

OCD = obsessive-compulsive disorder; SAD = social anxiety disorder; SD = standard deviation

***Table S2****: Differences in emotions associated with SLE between OCD and SAD groups*

|  | **OCD**  ***(n = 38)*** | | **SAD**  ***(n = 25)*** | |  |  |
| --- | --- | --- | --- | --- | --- | --- |
| **Emotion related to SLE** | **Mean** | **SD** | **Mean** | **SD** | ***Effect Size (r)*** | ***P-value*** |
| Fear/horror/  helplessness | 2.79 | 1.42 | 2.92 | 1.41 | *.05* | *.649* |
| Disgust | 0.84 | 1.35 | 1.12 | 1.62 | *.06* | *.556* |
| Rage | 2.08 | 1.42 | 2.12 | 1.67 | *.02* | *.880* |
| Guilt | 2.03 | 1.60 | 2.20 | 1.56 | *.05* | *.708* |
| Shame | 1.92 | 1.50 | 2.40 | 1.47 | *.16* | *.199* |
| Sadness | 3.13 | 1.12 | 2.84 | 1.55 | *.04* | *.729* |

OCD = obsessive-compulsive disorder; SAD = social anxiety disorder; SLE = stressful life event; SD = standard deviation
